# SG-APSIC1172: Mitigating the problems that arose in a ward with COVID-19 cases: Development of a “hot ward” tool kit for a potential COVID-19 outbreak

**DOI:** 10.1017/ash.2023.29

**Published:** 2023-03-16

**Authors:** Razali Mahdi, Somani Jyoti, Revathi Sridhar

**Affiliations:** National University Hospital, Singapore; National University Hospital, Singapore; National University Hospital, Singapore

## Abstract

**Objectives:** COVID-19 cases continue to climb in the community from the SARS-CoV-2 δ (delta) variant wave. To prepare for cases that may be nosocomial or detected late, the infection prevention team constructed a ‘hot ward’ tool kit to guide implementation of infection control measures. **Methods:** We engaged the NUH Facilities Management ventilation engineers to understand every ward’s mechanical ventilation setup. With this information, we created of “green” and “hot” zones within ward. After conducting assessments on individual wards, we created the “hot ward” tool kit: (1) 38 ward floor plans indicating ventilation setup, “green” zones, and “hot” zones; (2) a risk matrix to guide ward actions based on cycle threshold (Ct) value and duration of exposure; and (3) “hot ward” checklists. The tool kit was presented to infectious disease clinicians on the infection prevention team and senior nursing leaders for input and guidance. To ensure that these plans were practical, we conducted numerous site walks with HOD and ward nurse managers (ie, for the ICUs and psychiatric units). Finally, the tool kit was shared in a meeting with key stakeholders and senior leaders. It was also uploaded to the NUH COVID-19 quick-reference intranet page for easy staff access. **Results:** The tool kit was used by 2 general wards when cases of confirmed COVID-19 were detected among patients. Overall, the tool kit helped HOD and nurse managers with the immediate actions required and it provides useful guidance for the infection prevention team to assess and guide decisions regarding whether a ward lockdown is necessary. **Conclusions:** Although the guidance was useful, from the site walk we learned that the mechanical ventilation system of some wards is shared, making it challenging to prevent cross contamination between wards because any shared ventilation between unmasked areas can be pose a risk for both patients and staff. Additional measures were instituted to mitigate this risk.

